# Assessment of Serum Cytokine Levels in Keratoconus Patients

**DOI:** 10.3390/jcm14093179

**Published:** 2025-05-04

**Authors:** Noor Alqudah, Nosayba Al-Azzam, Leen El Taani, Abdallah Sharayah, Mohammad Al Qudah, Khawlah Mhedat, Suha Tahat

**Affiliations:** 1 Division of Ophthalmology, Department of Special Surgery, Faculty of Medicine, Jordan University of Science and Technology, Irbid 22110, Jordan; 2 Department of Physiology and Biochemistry, Faculty of Medicine, Jordan University of Science and Technology, Irbid 22110, Jordan; 3 Department of Nursing, King Abdullah University Hospital, Jordan University of Science and Technology, Irbid 22110, Jordan

**Keywords:** keratoconus, inflammation, biomarkers, cytokines, interleukin-6, TNF-α, interleukin-1β

## Abstract

**Background:** While keratoconus (KC) has traditionally been classified as a non-inflammatory corneal disorder, emerging evidence suggests that inflammatory processes may be involved in its pathogenesis. This study investigated whether systemic inflammation contributes to KC development by measuring serum levels of key pro-inflammatory cytokines in KC patients compared to healthy controls. **Methods:** This cross-sectional comparative study included 60 participants aged 18–30 and was divided into three equal groups: healthy controls, progressive KC, and stable KC. The levels of interleukin-6 (IL-6), tumor necrosis factor-alpha (TNF-α), and interleukin-1 beta (IL-1β) in the serum were measured through an enzyme-linked immunosorbent assay. KC severity was classified based on mean keratometry values. **Results:** There were no significant differences detected in serum IL-6, TNF-α, and IL-1β levels across groups (*p* > 0.05). Moreover, no significant correlations were found between systemic cytokine levels and KC severity categories (*p* > 0.05). **Conclusions:** Systemic levels of IL-1β, IL-6, and TNF-α do not significantly differ between KC patients and controls nor do they correlate with disease severity, reinforcing the hypothesis that KC involves primarily local inflammatory processes.

## 1. Introduction

Keratoconus (KC) is a progressive condition affecting the cornea, characterized by corneal thinning, a conical protrusion, and irregular astigmatism [1,2]. It often results in significant visual impairment [3]. The etiology of KC is multifactorial, arising from complex interactions between environmental triggers—like contact lens wear and eye-rubbing—and various endogenous factors [4,5,6,7]. These interactions contribute to corneal oxidative stress, driven by an amplified response to oxidative stressors [8]. This condition entails mitochondrial impairment and damage to mitochondrial DNA in individuals with a genetic predisposition [9].

Traditionally, KC corneal disorder has been classified as non-inflammatory because it lacks typical inflammatory features, such as neovascularization and the presence of infiltrating inflammatory cells [10]. However, this classification has been increasingly challenged by emerging evidence. Recent investigations have demonstrated elevated levels of inflammatory molecules in the tear film of KC patients, including interleukins (IL-1, IL-5, IL-6, IL-8) and tumor necrosis factors (TNF-α and TNF-β), when compared to healthy controls [11]. Furthermore, the magnitude of these elevations has been shown to correlate with disease severity, suggesting that chronic inflammation may play a significant role in KC pathogenesis [12,13].

The distinction between systemic and localized inflammation in KC has important implications for disease management and therapeutic development. If systemic inflammatory processes are involved in KC, targeted anti-inflammatory interventions could be developed to slow or halt disease progression. This approach would represent a significant advancement in KC management, focusing primarily on visual rehabilitation rather than addressing underlying pathophysiological mechanisms [14].

To address this knowledge gap, our study aimed to assess serum levels of key pro-inflammatory cytokines—specifically interleukin one beta (IL-1β), interleukin 6 (IL-6), and tumor necrosis factor-alpha (TNF-α) in KC patients compared to healthy controls. These particular cytokines were selected based on their established roles in promoting inflammation, extracellular matrix degradation, and tissue remodeling—processes fundamental to KC development [15,16]. By evaluating systemic cytokine expression patterns, we aimed to clarify whether KC disease involves systemic inflammatory pathways or is confined to localized cornea and tear film inflammation, as recent studies have shown.

## 2. Methods and Materials

### 2.1. Study Design and Settings

This cross-sectional comparative study was conducted at the ophthalmology outpatient clinics at King Abdullah University Hospital, Jordan, between March 2024 and October 2024. The research protocol adhered to the tenets of the Declaration of Helsinki and received approval from the Institutional Review Board (IRB No. 2023/845). Written informed consent was obtained from all participants before enrollment and biological sample collection.

### 2.2. Participant Selection and Categorization

Sixty participants aged 18 to 30 years were enrolled in the study and stratified into three distinct groups: healthy controls (n = 20), progressive KC (n = 20), and stable KC (n = 20). The categorization was based on comprehensive ophthalmological evaluation and corneal topographical assessment.

#### 2.2.1. Inclusion Criteria

Participants aged 18 to 30 with clinical and topographic manifestations of KC were included in the patient groups.

Age–gender-matched subjects with normal corneal topography were recruited for the control group.

Progressive KC was defined by documented evidence of significant corneal topographic alterations as determined by Pentacam imaging (Oculus Optikgeräte, Wetzlar, Germany). Specifically, progression was characterized by either an increase of 1.00 diopter (D) or greater in maximum keratometry (Kmax) at the corneal apex within a 12-month period or a mean reduction in corneal thickness of 5% or more during the same timeframe.

The stable KC group comprised cases who had undergone corneal cross-linking (CXL) procedure at least one year before study enrollment and demonstrated no evidence of disease progression according to the aforementioned parameters.

The extent of KC was categorized based on the average keratometry (K mean) measurements: mild (K mean < 48), moderate (K mean ≥ 48 and <53), and severe (K mean ≥ 53).

#### 2.2.2. Exclusion Criteria

Individuals were excluded as follows: existence of concomitant corneal pathologies; history of ocular surgery (apart from CXL in the stable KC group); prior diagnosis of active systemic or ocular inflammation, connective tissue disorders, diabetes mellitus, glaucoma, rheumatological conditions, uveitis; administration of systemic or topical anti-inflammatory medications within one month prior to enrollment (only artificial tears were permitted); history of hepatic, renal, metabolic, hematological, or immunological disorders; documented infection within one month preceding blood sample collection; psychiatric disorders; use of corticosteroids; pregnancy; and lactation. These stringent exclusion criteria were implemented to minimize confounding variables that might influence inflammatory biomarker profiles.

### 2.3. Laboratory Investigations

#### 2.3.1. Blood Sample Collection and Processing

Every participant had venous blood samples taken in 5 mL tubes containing gel and clot activator (AFCO, Amman, Jordan). After 30 min of coagulation at room temperature, the specimens were centrifuged for 5 min at 4500 rpm. The resulting serum was carefully aspirated, aliquoted into Eppendorf tubes, and stored at −80 °C until cytokine analysis was performed.

#### 2.3.2. Cytokines Measurement

Serum concentrations of three pro-inflammatory cytokines, TNF-α, IL-6, and IL-1β, were quantified using an enzyme-linked immunosorbent assay (ELISA) methodology. The assays were conducted in accordance with the manufacturer’s protocols. TNF-alpha levels were determined using the Invitrogen ELISA kit (Cat. No. KHC3011). IL-6 and IL-1β concentrations were measured using Abcam ELISA kits (Cat. No. ab46042 and Cat. No. ab46052, respectively).

### 2.4. Sample Size

The sample size calculation was performed by G*Power 3.1.9.2 (Universitat Kiel, Germany). We performed a pilot study (5 cases in each group), and we found that the mean (±SD) of IL-6 was 8.13 ± 3.8 ng/mL in the healthy group, 6.64 ± 0.64 ng/mL in the progressive keratoconus group, and 11.49 ± 8.92 ng/mL in the stable keratoconus group. The sample size was based on the following considerations: a 0.451 effect size, 95% confidence limit, the 80% power of the study, and group ratio of 1:1:1, and 3 cases were added to each group to overcome dropout. Therefore, we recruited 20 patients in each group.

### 2.5. Statistical Analysis

Statistical analysis was performed by SPSS v27 (IBM©, Chicago, IL, USA). The Shapiro–Wilks test and histograms were used to evaluate the normality of the data distribution. Quantitative parametric data were presented as mean and standard deviation (SD) and were analyzed by ANOVA (F) test with post hoc test (Tukey). Quantitative non-parametric data were presented as the median and interquartile range (IQR) and were analyzed by the Kruskal–Wallis test with Mann–Whitney test to compare each group. Qualitative variables were presented as frequency and percentage (%) and analyzed using the Chi-square test—spearman rank correlation equation for non-normal variables/non-linear monotonic relation. A two-tailed *p* value < 0.05 was considered statistically significant.

## 3. Results

Our demographic analysis revealed that the three study groups (healthy controls, progressive KC, and stable KC) were generally well-matched, with a few notable differences. The mean age varied significantly across groups (*p* = 0.033), with stable KC patients being slightly older (25.4 ± 3.97 years) than both progressive KC patients (22.15 ± 3.34 years) and healthy controls (23.4 ± 4.18 years). Gender distribution was balanced across all three groups (50% male, 50% female). Body mass index (BMI) showed a trend toward higher values in stable KC patients (28.20 ± 7.84 kg/m^2^) compared to both progressive patients (24.75 ± 3.62 kg/m^2^) and healthy controls (25.69 ± 4.46 kg/m^2^), though this difference did not reach statistical significance (*p* = 0.143). Smoking status varied numerically across groups (45% in controls, 63% in progressive KC, and 75% in stable KC), but was not statistically significant (*p* = 0.144). Occupational status exhibited some variation, with fewer employed individuals in the progressive KC group (20%) than stable KC (40%) and healthy controls (50%), though this difference was not statistically significant (*p* = 0.134) (Table 1).

**Figure 1:**
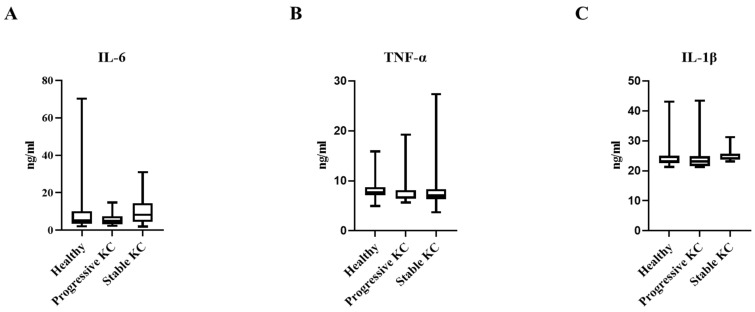
Serum cytokine concentration levels for (**A**) IL-6, (**B**) TNF-α, (**C**) IL-1β in stable keratoconus, progressive keratoconus, and healthy control groups.

Analysis of inflammatory markers revealed statistically insignificant patterns across the three study populations. There was no statistically significant difference in IL-6 levels among healthy controls [5.12 ng/mL (IQR 3.85–10.16)], progressive KC patients [4.9 ng/mL (3.46–6.9)], and stable KC patients [8.18 ng/mL (4.54–13.26)] (*p* = 0.294). TNF-α concentrations were remarkably consistent across groups, with mean values of 8.22 ± 2.55 ng/mL, 7.93 ± 3.17 ng/mL, and 8.57 ± 5.29 ng/mL in healthy controls, progressive KC, and stable KC, respectively (*p* = 0.872). Similarly, IL-1β levels remained virtually identical across all three groups (24.78 ± 4.99 ng/mL, 24.58 ± 4.99 ng/mL, and 25.18 ± 2.32 ng/mL, respectively; *p* = 0.903) (Table 2 and Figure 1).

When stratifying the KC patients by disease severity, we observed a counterintuitive trend in IL-6 levels, with concentrations progressively decreasing with increasing disease severity (9.0240 ± 7.6352 ng/mL in mild, 7.0017 ± 5.8027 ng/mL in moderate, and 6.342 ± 3.3310 ng/mL in severe KC), although this pattern failed to reach statistical significance (*p* = 0.593). IL-1β concentrations remained remarkably stable across all severity categories (24.9407 ± 4.4240 ng/mL, 24.9618 ± 2.8323 ng/mL, and 24.247 ± 2.2336 ng/mL for mild, moderate, and severe disease, respectively; *p* = 0.941). Similarly, TNF-α levels exhibited a non-significant trend toward lower concentrations in more severe disease (8.9026 ± 5.2466 ng/mL in mild, 6.9365 ± 0.8192 ng/mL in moderate, and 7.3175 ± 0.6868 ng/mL in severe KC; *p* = 0.546). These results support the hypothesis that KC progression is predominantly driven by localized corneal inflammation rather than systemic inflammatory mechanisms (Table 3).

Correlation analysis further confirmed the lack of a significant association between inflammatory cytokine levels and KC severity. Spearman’s correlation coefficients revealed weak negative correlations for IL-6 (r = −0.108, *p* = 0.504) and TNF-α (r = −0.082, *p* = 0.613), and a weak positive correlation for IL-1β (r = 0.081, *p* = 0.615). The negligible magnitude and lack of statistical significance of these correlations suggest that systemic levels of these inflammatory markers do not serve as reliable indicators of KC severity or progression status, emphasizing local inflammation (Table 4).

## 4. Discussion

KC is a progressive, non-inflammatory condition of the cornea marked by bilateral yet asymmetrical thinning, which results in corneal protrusion, irregular astigmatism, and compromised vision [17,18,19]. While the exact origins and development of KC remain unclear, genetic and external influences are thought to contribute, and growing research indicates that inflammatory processes may significantly influence the disease’s initiation and advancement [20,21].

The current study found that there was an absence of statistically significant differences in serum levels of IL-6 among healthy controls, progressive KC patients, and stable KC patients. This observation contradicts some existing literature that suggests elevated IL-6 levels in tear samples from KC patients compared to healthy controls [22,23]. Shetty et al. [24] found dramatically elevated IL-6 levels in tears of KC patients (2413 ± 489 pg/mL) compared to controls (49 ± 21 pg/mL, *p* = 0.004). Similarly, Peyman et al. [25] reported significantly higher tear IL-6 levels in KC patients (103.22 ± 51.94 pg/mL) than in controls (26.77 ± 8.16 pg/mL, *p* < 0.001). D’Souza et al. [26] further demonstrated that IL-6 levels in tear fluid progressively increased with KC severity, ranging from approximately 15 pg/mL in early grades to 20 pg/mL in advanced cases.

Our study found that systemic TNF-α and IL-1 β levels remained consistent across all study groups, with no substantial variations observed between healthy controls, progressive KC, and stable KC cases. This observation contrasts with several studies examining tear cytokine levels in KC patients. Zhang et al. [10] conducted a meta-analysis of seven studies involving 374 participants. They reported significantly elevated levels of both TNF-α (SMD = 1.75, 95% CI 0.66–2.83, *p* = 0.002) and IL-1β (SMD = 1.93, 95% CI 0.22–3.65, *p* = 0.03) in tears of KC cases than controls. Similarly, Shetty et al. [24] demonstrated substantially increased tear levels of TNF-α (806 ± 168 pg/mL vs. 96 ± 49 pg/mL, *p* = 0.007) and IL-1β (2474 ± 486 pg/mL vs. 24 ± 21 pg/mL, *p* = 0.001) in KC cases than controls.

Balasubramanian et al. [27] provided mechanistic insights by demonstrating that experimental eye rubbing in normal volunteers significantly increased tear levels of IL-6 (1.24 ± 0.98 vs. 2.02 ± 1.52 pg/mL, *p* = 0.004) and TNF- α (1.16 ± 0.74 vs. 1.44 ± 0.66 pg/mL, *p* = 0.003).

On the other hand, Pinheiro-Costa et al. [28] explored the relationship between serum inflammatory mediators and choroidal thickness in KC patients versus control subjects; they reported modest but statistically insignificant elevations in serum IL-1, IL-6, and TNF-α levels in KC patients compared to healthy controls. This finding aligns with our results and suggests that these cytokines, when measured systemically, may not serve as reliable biomarkers for KC diagnosis or progression monitoring.

Our study demonstrated no notable correlation between systemic cytokine (IL-6, TNF- α, and IL-1β) levels and KC severity categories. This finding reinforces the hypothesis that KC progression is predominantly driven by localized corneal inflammation rather than systemic inflammatory mechanisms. This observation also contrasts with studies examining tear cytokine correlations with KC severity. D’Souza et al. [26] reported that TNF-α levels in tear fluid exhibited a significant positive correlation with keratometry indices, with correlation coefficients ranging from r = 0.388 (K1) to r = 0.410 (Kmax). Similarly, IL-1β levels correlated positively with keratometry indices, with values between r = 0.307 (K1, near significance) and r = 0.345 (Kmax, *p* = 0.027). Peyman et al. [25] also demonstrated that disease severity in KC patients was strongly correlated with increased tear IL-6 and TNF-α levels (*p* < 0.001).

Krok et al. [29] provided further evidence of the association involving local inflammatory markers and KC severity, reporting that moderate and severe KC cases exhibited significantly higher tear levels of IL-1β, IL-10, IFN-γ, and TNF-α than mild cases (*p* < 0.05). Additionally, they found that TNF-α, IFN-γ, and IL-6 levels positively correlated with keratometry values preoperatively and 12 months post-CXL (*p* < 0.05, r > 0).

It is essential to differentiate between tear (local) and serum (systemic) cytokine measurements when investigating inflammatory processes in KC. The present study specifically examined systemic cytokine levels, whereas most previous research has focused on tear fluid analysis. Since eye rubbing is a well-established risk factor for KC development and progression, the findings suggest that mechanical trauma may induce a localized inflammatory cascade in predisposed individuals [30]. This interpretation aligns with the findings of García-Onrubia et al. [31], who reported distinct cytokine profiles in tears and serum in patients with uveitis, indicating that tear cytokines may reflect localized ocular inflammation independently of systemic cytokine levels.

This research is subject to certain limitations. The number of participants was comparatively limited, which may restrict the applicability of the results to a broader population. Additionally, the study focused solely on systemic cytokine levels, while previous research highlights the importance of local (tear) cytokine profiles in KC. Confounding factors such as environmental exposures and genetic predispositions were not fully accounted for, which may influence inflammatory biomarker levels. Additionally, the study did not concurrently analyze tear cytokine levels, which have been strongly associated with KC pathogenesis in prior research.

Multi-center studies with larger cohorts are required to validate these findings. Incorporating paired serum and tear samples in future research could clarify whether systemic inflammation indirectly affects local corneal processes or if keratoconus progression is solely driven by compartmentalized ocular inflammation. Further investigations should also assess additional biomarkers, including IL-8, MMP-9, and IFN-γ, in both serum and tear fluid.

## 5. Conclusions

This study found no significant correlation between systemic cytokine levels (IL-6, TNF-α, or IL-1β) and KC pathogenesis or severity, indicating that systemic inflammation does not play a significant role in disease progression. These results emphasize that KC is primarily driven by localized corneal inflammation rather than systemic cytokine activity, reinforcing the need for targeted local anti-inflammatory therapies.

## Figures and Tables

**Table 1:** Demographic and clinical characteristics between studied groups.

Characteristics		Healthy (n = 20)	Progressive KC (n = 20)	Stable KC (n = 20)	*p* Value
Age		23.4 ± 4.18	22.15 ± 3.34	25.4 ± 3.97	0.033
Gender					
	Male	10 (50%)	10 (50%)	10 (50%)	1
	Female	10 (50%)	10 (50%)	10 (50%)	
Body mass index (kg/m^2^)		25.69 ± 4.46	24.75 ± 3.62	28.20 ± 7.84	0.143
Smoking		9 (45%)	12 (63%)	15 (75%)	0.144
Occupation					
Employed		10 (50%)	4 (20%)	8 (40%)	0.134
Self-employed		0 (0%)	1 (5.0%)	1 (5.0%)	
Student		10 (50%)	11 (55%)	6(30%)	
Unemployed		0 (0%)	4 (20%)	5(25%)	

Data are presented as mean ± SD or count frequency (%). KC: keratoconus.

**Table 2:** Inflammatory cytokine profiles between studied groups.

	Healthy (n = 20)	Progressive KC (n = 20)	Stable KC (n = 20)	*p* Value
IL-6 (ng/mL)	5.12 (3.85–10.16)	4.90 (3.46–6.9)	8.18 (4.54–13.26)	0.294
TNF-α (ng/mL)	8.22 ± 2.55	7.93 ± 3.17	8.57 ± 5.29	0.872
IL-1B (ng/mL)	24.78 ± 4.99	24.58 ± 4.99	25.18 ± 2.32	0.903

Data are presented as mean ± SD or median (IQR). KC: keratoconus.

**Table 3:** Cytokine levels in relation to keratoconus severity.

Severity Category	IL-6 Level (ng/mL)	IL-1β Level (ng/mL)	TNF-α Level (ng/mL)	*p* Value
Mild	9.02 ± 7.64	24.94 ± 4.42	8.90 ± 5.25	0.593
Moderate	7 ± 5.80	24.96 ± 2.83	6.94 ± 0.82	0.941
Severe	6.34 ± 3.33	24.25 ± 2.23	7.32 ± 0.69	0.546

Data are presented as mean ± SD.

**Table 4:** Correlation between cytokines and keratoconus severity.

Cytokine	Spearman’s Correlation	*p* Value
IL-6	−0.108	0.504
TNF-α	−0.082	0.613
IL-1β	0.081	0.615

## Data Availability

Due to privacy and ethical restrictions, the datasets used and analyzed during the current study are available from the corresponding author upon reasonable request.
